# Retraction: The iron group transition-metal (Fe, Ru, Os) coordination of Se-doped graphitic carbon (Se@g-C_3_N_4_) nanostructures for the smart therapeutic delivery of zidovudine (ZVD) as an antiretroviral drug: a theoretical calculation perspective

**DOI:** 10.1039/d5ra90039e

**Published:** 2025-04-04

**Authors:** Favour A. Nelson, Hitler Louis, Innocent Benjamin, Rawlings A. Timothy

**Affiliations:** a Computational and Bio-simulation Research Group, University of Calabar Calabar Nigeria louismuzong@gmail.com; b Department of Microbiology, University of Calabar Calabar Nigeria; c Department of Pure and Applied Chemistry, University of Calabar Calabar Nigeria; d Centre for Herbal Pharmacology and Environmental Sustainability, Chettinad Hospital and Research Institute, Chettinad Academy of Research and Education Kelambakkam 603103 Tamil Nadu India

## Abstract

Retraction of ‘The iron group transition-metal (Fe, Ru, Os) coordination of Se-doped graphitic carbon (Se@g-C_3_N_4_) nanostructures for the smart therapeutic delivery of zidovudine (ZVD) as an antiretroviral drug: a theoretical calculation perspective’ by Favour A. Nelson *et al.*, *RSC Adv.*, 2023, **13**, 34078–34096, https://doi.org/10.1039/D3RA06885D.

The Royal Society of Chemistry hereby wholly retracts this *RSC Advances* article due to concerns with the reliability of the data.

The molecular dynamics (MD) simulations have not been described satisfactorily with mistakes throughout, such as time used of 1 ps. In addition, some of the results are inconsistent with the statements made by the authors, such as the temperatures shown in Fig. 7.

The authors have not been able to provide the input or trajectory file generated from the MD simulations.

Given the significance of these concerns, the Editor has lost confidence that the findings presented in this paper are reliable.

The authors were informed but have not responded to any correspondence regarding the retraction.

Signed: Laura Fisher, Executive Editor, *RSC Advances*

Date: 28th March 2025

